# Adiponectin Gene Polymorphisms and Possible Susceptibility to Metabolic Syndrome among the Sudanese Population: A Case-Control Study

**DOI:** 10.1155/2023/5527963

**Published:** 2023-04-27

**Authors:** Altaf S. Mosad, Ghada A. Elfadil, Alsadig Gassoum, Khanssa M. Elamin, Nazik Elmalaika Obaid Seid Ahmed Husain

**Affiliations:** ^1^Department of Clinical Chemistry, College of Medical Laboratory Science, Sudan University of Science and Technology, Khartoum, Sudan; ^2^National Center of Neurological Sciences, Khartoum, Sudan; ^3^Faculty of Medicine, University of Mashreq, Khartoum, Sudan; ^4^Department of Pathology, Faculty of Medicine and Health Sciences, Omdurman Islamic University, Khartoum, Sudan

## Abstract

**Results:**

There is a significant difference in genotypic frequencies of the rs266729, rs2241766, and rs1501299 SNPs and allele frequencies (*P*  < 0.05) between the MetS patients and non-MetS group. MetS patients had a significantly higher serum triglycerides (TG), total cholesterol (TC), and low-density lipoprotein-cholesterol (LDL-C) in the GG genotype of rs2241766 (*P*  < 0.05). Additionally; the TT genotype of rs1501299 had higher SBP, serum TG, TC, and LDL-C (*P*  < 0.05). Multivariate logistic regression analysis showed hypertension, hyperglycemia, BMI, WC, serum TG, ADIPOQ rs2241766 (TG allele), and ADIPOQ rs1501299 (GT allele) had independently predicted the incidence of metabolic syndrome in the Sudanese population. The three investigated SNPs of ADIPOQ were in a moderate linkage disequilibrium (LD) connection according to the LD measures (D' = 0.54, 0.62, and 0.69, respectively). The CTT, CGG, and GTG haplotypes, which consist of three alleles of −11377C > G, +45T > G, and +276G > T, were shown to report 1.788-, 1.622,- and 1.641-fold risks toward MetS susceptibility in Sudanese's population, respectively.

**Conclusion:**

Along with clinical and biochemical signs, the ADIPOQ gene's genetic variants (rs266729, rs2241766, and rs1501299), CTT, CGG, and GTG haplotypes are connected to the MetS risk among the Sudanese population.

## 1. Introduction

The most recent pandemic of disorders that has affected humans is metabolic syndrome (MetS). Since obesity and type 2 diabetes (T2D) are included in the MetS category, it has ensnared millions worldwide [1, 2]. MetS, as determined by the Adult Treatment Panel III criteria of the National Cholesterol Education Program (NCEP ATP III), was identified when at least three of the following factors were present: abdominal obesity (WC ≥ 102 cm, ≥88 cm in men and women, respectively), elevated TG (≥150 mg/dL), reduced HDL-C (<40 mg/dL, <50 mg/dL in men and women), increased blood pressure (≥130/85 mmHg), and elevated fasting plasma glucose (≥110 mg/dL) [3, 4]. MetS was present in 16.6% of the Sudanese population [4]. Because of the increased frequency of MetS and its impact on public health, early detection and management of MetS are crucial to preventing the onset of T2D, cardiovascular disease, and other consequences.

The ADIPOQ gene, situated on chromosome 3q27 [5, 6] and only expressed in human adipose tissue, is responsible for producing the plasma protein adiponectin [7, 8]. T2D, obesity, and metabolic syndrome have all been linked to the 3q27 gene [9, 10]. Numerous researchers have looked into the adiponectin gene area in various populations to find genetic variations that substantially influence the pathophysiology of obesity, metabolic syndrome (MetS), diabetes, and related complications [11].

Three exons make up the ADIPOQ obesity-associated variants, and the rs2241766, rs266729, and rs1501299 were frequently studied [8]. The minor allele G of the SNP rs266729, which is situated in the gene's promoter region, changes the sequence of the binding site (SP1), resulting in a loss of the SP1 binding effect and perhaps reducing the transcription activity of the adiponectin promoter [10]. The SNP rs2241766, found in exon 2 of the gene, may have polymorphic effects on the stability or shearing of precursor mRNA or the level of proteins. The ADIPOQ gene's second intron has the SNP rs1501299 and studies suggest that this polymorphism may impact how well the exon next to it functions [9]. The polymorphism of these two locations influences the body's insulin sensitivity and stimulates the development of T2D [9]. Therefore, this study aimed to ascertain the genotypes, haplotypes, and allelic distribution of the ADIPQ gene polymorphisms (rs266729, rs2241766, and rs1501299) and its relationship to MetS in Sudanese population. Additionally, ADIPQ gene SNPs based on BMI, HbA1c, and the lipid profile in the Sudanese people are investigated.

## 2. Methodology

Four hundred and twenty middle-aged adult Sudanese participants (210 had MetS and 210 non-MetS) were recruited for this community-based case-control study between July 2019 and July 2021. After getting ethical approval from the Research Committee of the Sudan University of Science and Technology (DSR-IEC-04-08), all participants received written informed consent forms after being told of the study's purpose. Patients with liver illness, renal failure, autoimmune disorders, and corticosteroid medication and patients with incompletely documented data were excluded from this study based on their medical records. Participants were divided into two groups (MetS and non-MetS), matched according to age and gender and based on whether they had MetS, as determined by the NCEP ATP III criteria.

### 2.1. Data Collection and Analysis

Patients' weights were measured using a weight balance, their heights were measured using a meter, and their waists were measured using stretch-resistant tape between the bottom edge of the lower rib and the iliac crest. A mercury sphygmomanometer was used to check the patient's blood pressure (SBP and DBP). Five milliliters of fasting venous blood was drawn in the morning and divided into two containers: a whole blood container (EDTA) for HbA1c measurement and DNA extraction and a plain container for serum TG, TC, LDL-C, and HDL-C measurements. Automated spectrophotometers (Mindray-BA-88A) and ichroma (Boditech-I-CHROMA Reader) were used to measure serum TG, TC, LDL-C, HDL-C, and HbA1c, respectively. BMI was calculated by dividing body weight/kg by height/m^2^ [12].

### 2.2. DNA Extraction

Genomic DNA was extracted from whole blood using a guanidine extraction protocol [13]. The quality of DNA was assessed using 2.0% gel electrophoresis.

### 2.3. Screening of ADIPOQ SNPs

**Figure 1:**
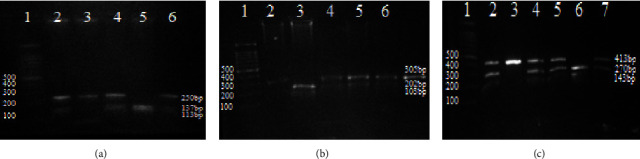
Genotypes for the ADIPOQ SNPs are as follows: (a) (rs266729), (b) (rs2241766), and (c) (rs1501299). In image (a), L1 stands for a 100 bp ladder; L3 stands for a wild genotype (250 bp); L2, L4, and L6 stand for heterozygous genotypes (250 bp, 137 bp, and 113 bp); and L5 for a mutant genotype (137 bp and 113 bp). In image (b), L1 denotes a 100 bp ladder; L2 denotes a wild genotype (305 bp); L4, L5, and L6 denote heterozygous genotypes (305 bp, 202 bp, and 103 bp); and L3 denote mutant genotypes (202 bp and 103 bp). In image (c), L1 stands for the 100 bp ladder; L6 stands for the wild genotypes (270 bp and 143 bp); L2, L4, L5, and L7 stand for the heterozygous genotypes (413 bp, 270 bp, and 143 bp); and L3 stands for the mutant genotype (413 bp).

PCR-RFLP was used to amplify and genotype ADIPOQ variants −11377C/G (rs266729), +45T/G (rs2241766), and +276G/T (rs1501299). The amount of the PCR reaction mixture was 20 *μ*L, and the annealing temperatures varied depending on the primers (Table 1). A 2.0% agarose gel stained with ethidium bromide was used to test the amplified products for electrophoresis. Table 1 shows the specification of the restriction enzymes used in this study, obtained from Fermentas, Thermo Fisher Scientific Inc., USA. Following the manufacturer's recommendations, 1U of the relevant restriction enzyme was used to digest 5 *μ*L of the amplified products in a total reaction volume of 10 *μ*L. The 100 base pair DNA ladder and the digestion products were resolved on 2.0% agarose gels stained with ethidium bromide and seen under a UV transilluminator (SYNGENE-InGeniusL). Gel photos of the ADIPOQ gene are shown in Figure 1

## 3. Statistical Analysis

Statistical Package for Social Sciences (SPSS; Chicago, USA, version 20.0) was used for all statistical analyses. The one-sample Kolmogorov–Smirnov test was used to detect the normal distribution of data for baseline parameters. The Hardy–Weinberg equilibrium (HWE) was evaluated for all the SNPs in patients and controls by comparing the observed and expected frequencies of the genotypes using the chi-squared test. An independent *t*.test was used to analyze baseline parameters. The distribution of the genotypes and allele frequencies of ADIPOQ SNPs for patients and control were compared using the chi-squared test with two × two contingency tables using GraphPad Prism 6 software. The association of SNPs with other parameters was performed using the one-way ANOVA test. The association of the ADIPOQ SNPs with the risk of MetS was assessed by the odds ratio (OR) with 95% confidence intervals (CI) and logistic regression to predict risk factors. Linkage disequilibrium analysis of the three adiponectin gene SNP loci haplotypes was performed using SHEsis online software (http://analysis.bio-x.cn).

For all statistical tests for bilateral comparisons, *P* ≤ 0.05 was considered statistically significant.

## 4. Results

**Figure 2:**
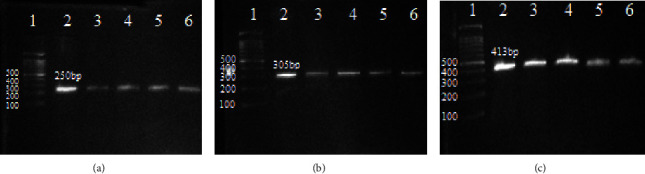
PCR amplification for the ADIPOQ SNPs (a) (rs266729), (b) (rs2241766), and (c) (rs1501299). L1 stands for a 100 bp ladder, while L2–L6 indicate amplified PCR products of 250 bp in image (a), 305 bp in image (b), and 413 bp in image (c).

Table 2 summarizes the clinical and biochemical characteristics of the participants. MetS patients showed substantially greater BMI, WC, SBP, and DBP when compared to non-MetS individuals (*P*=0.001). In addition, compared to non-MetS individuals, MetS patients had considerably higher HbA1c, serum TG, TC, and LDL-C levels while having significantly lower HDL-C levels. Genetic analysis showed that the ADIPOQ gene polymorphisms −11377C/G, +45T/G, and +276G/T were detected in all the participants. PCR products of C/G SNP (rs266729), T/G SNP (rs2241766), and G/T SNP (rs1501299) were 250 bp, 305 bp, and 413 bp, respectively (Figure 2). Restriction digestion was carried out following amplification. For the rs266729 genotype, the wild genotype (CC) produced a single band of 250 bp; the heterozygous genotype (CG) had three fragments of 250 bp, 137 bp, and 113 bp; and the homozygous mutant genotype (GG) produced two bands of 137 bp and 113 bp. For rs2241766, the wild genotype (TT) had a single band 305 bp; the heterozygous genotype (TG) produced three fragments 305 bp, 202 bp, and 103 bp; and the homozygous mutant genotype (GG) produced two bands 202 bp and 103 bp. For rs1501299, the wild genotype (GG) was digested and made two bands, 270 bp and 143 bp; the heterozygous genotype (GT) had three rounds, 413 bp, 270 bp, and 143 bp; and the homozygous mutant genotype produced a single band, 413 bp (Figure 1).  Table 3 summarizes the genotypes and allele frequencies of ADIPOQ SNPs (rs266729, rs2241766, and rs1501299).

In the present study, the frequencies of mutant alleles −11377C/G (rs266729), +45T/G (rs2241766), and +276G/T (rs1501299) were considerably more significant in MetS participants (OR = 2.150, 2.293, and 2.228, respectively). Furthermore, one-way ANOVA and post hoc multiple comparison analysis of +45T/G (rs2241766) showed significantly higher serum triglycerides (TG), total cholesterol (TC), and LDL-C level in the GG genotype (*P*  < 0.05) than in TT and TG genotypes. The +276G/T (rs1501299) increased the levels of SBP, serum triglycerides (TG), total cholesterol (TC), and low-density lipoprotein (LDL-C) in the TT genotype compared to GG and GT genotype, *P*  < 0.05 (Table 4). The relationship between the genotypes −11377C > G (rs266729), +45T > G (rs2241766), and +276G > T (rs1501299) and metabolic elements with the risk of MetS are listed in Table 5.

Using logistic regression univariate analysis, the significant risks of metabolic syndrome were hypertension, hyperglycemia, BMI, WC, serum TG, TC, LDL-C, HDL-C, ADIPOQ rs266729 (CG, GG), ADIPOQ rs2241766 (TG, GG), and ADIPOQ rs1501299 (GT, TT). However, it was found that hypertension, hyperglycemia, BMI, WC, serum TG, ADIPOQ rs2241766 (TG), and ADIPOQ rs1501299 (GT) can independently predict the occurrence of metabolic syndrome in healthy Sudanese adults when using multivariate logistic regression analysis. The distribution of the research population's haplotype frequencies and linkage disequilibrium (LD) are displayed in Table 6. The three ADIPOQ SNPs (rs266729, rs2241766, and rs1501299) were associated with moderate LD levels (Dʹ = 0.54, 0.62, and 0.69, respectively). According to the LD measurements, CTT, CGG, and GTG haplotypes of 11377C > G, +45T > G, and +276G > T SNPs showed ORs 1.788, 1.622, and 1.641, respectively, with high risks towards MetS susceptibility.

## 5. Discussion

Metabolic syndrome (MetS) is a recognized risk factor for the onset of cardiovascular disease and other degenerative illnesses, which are the major causes of morbidity and mortality [2, 10, 11]. The adiponectin gene has been identified as a human adiposity marker after significant research into the relationships between genetic differences in the adiponectin gene and several obesity measurements and metabolic syndrome in numerous ethnic cultures [11]. This study was carried out to determine the relationship between the risk of MetS in the included studies and the polymorphisms in the ADIPOQ gene (SNP-11377C > G, SNP +45T > G, and SNP +276G > T). LD analysis revealed that the three loci rs266729, rs2241766, and rs1501299 were in a moderate LD block. For the first time, rs266729, rs2241766, and rs1501299 were discovered in a haplotype block and connected to MetS in Sudan. For the first time, all of our results point to the possibility that the haplotypes ADIPOQ CTT, CGG, GTG, CGT, and GTT were linked to MetS. Further, CTT was more significantly linked to MetS, and haplotype CTG is genetically protective against MetS. Additionally, in the Sudanese population, ADIPOQ-11377C > G (rs266729), +45T > G (rs2241766), and +276G > T (rs1501299) were all significantly linked with MetS. There were considerably more carriers of the CG and GG genotypes in the MetS group (22.8% and 6.2%) than in the non-MetS group (15.2% and 1.5%) in this study's analysis of the genotype distribution of −11377C > G (rs266729) among MetS patients and non-MetS controls. It is also shown that the rs266729G allele raises the probability of developing metabolic syndrome in the research participants by 2.150 times (*P*=0.001). Our results are in line with a study by Garacia Robles et al. that suggests the minor allele G in the ADIPQ gene polymorphism rs266729 constitutes a risk factor for the development of MetS [10], and a study by Smetnev et al. that reports the SNP rs266729 of the ADIPOQ gene are significantly associated with cardiovascular and metabolic diseases [14].

Additionally, this study demonstrated a statistically significant difference in the genotype distribution of +45T > G (rs2241766) between MetS cases and non-MetS controls (*P*=0.03). There were significantly more carriers of the TG and GG genotypes in the MetS group (27.6% and 4.8%) than in the non-MetS group (16.2% and 1.0%). It is also discovered that the rs2241766G allele increases the risk of developing metabolic syndrome in the study participants by 2.293 folds (*P*=0.001). Our results are supported by research by Zahary et al. that indicates the genetic polymorphisms at the ADIPOQ +45T > G (rs2241766) loci enhanced the risk factor for the onset of MetS [15]. When looking at the relationship between the +276G > T (rs1501299) variation and MetS, it was shown that the T-minor allele contributed to a 2.228 fold (*P*=0.001) increased risk of susceptibility to metabolic syndrome (MetS) in the current population. Our results are in line with research by Zahary et al., which suggests the genetic variants at the ADIPOQ +276G > T (rs1501299) loci enhanced the risk factor for developing MetS [15]. These significant associations persisted after the adjustments to hypertension, hyperglycemia, BMI, WC, serum TG, HDL-C, ADIPOQ rs2241766 (TG), and ADIPOQ rs1501299 (GT).

Moreover, our results are consistent with the information provided by Kaur et al. They demonstrated that the ADIPOQ +276G > T (rs1501299) polymorphism might be clinically useful in determining the risk of obesity and MetS [11]. The results of the current investigation showed that the mutant genotypes −11377C > G (rs266729), +45T > G (rs2241766), and +276 G> T (rs1501299) were not significantly associated with any of the obesity indices (BMI and WC). Our findings are in line with research by Ogundele et al. that found no correlation between +276G > T (rs1501299) and obesity indicators in the Nigerian population [8]. Our results, however, are the exact reverse of those obtained by Cui et al., who found a connection between central obesity and the ADIPOQ gene [7].

Furthermore, our study found no correlation between the genotypes of −11377C/G (rs266729), +45T/G (rs2241766), and +276G/T (rs1501299) and the risk factors for the metabolic syndrome (HbA1c, SBP, DBP, serum TG, TC, LDL-C, and HDL-C), except the +45T/G (rs2241766) which proofed to be higher in serum triglycerides (TG), total cholesterol (TC), and LDL-C in the GG genotype (*P*  < 0.05). Additionally, it showed that the +276G/T (rs1501299) SNP appeared to have higher SBP, serum triglycerides (TG), total cholesterol (TC), and low-density lipoprotein (LDL-C) in the TT genotype (*P*  < 0.05).

From another point of view, it is critical to assess if the polymorphisms affect the alteration of MetS components and other obesity-related disorders following a change in lifestyle, such as dieting. De Luis et al. demonstrated that in this situation the CC genotype of the ADIPQ-11377C > G polymorphism is connected to increase in adiponectin levels and decrease in total cholesterol, LDL-C, glucose, and insulin levels as well as the homeostasis model assessment for insulin resistance after weight loss [16]. All parameters showed the same improvement after a diet high in polyunsaturated fat [17]. One interventional study of 60 severely obese patients evaluated 32 months after bariatric surgery revealed that C-allele homozygotes decreased BMI and the lipid profile in obese people [18].

Our haplotype analysis revealed that the MetS participants had significantly higher frequencies of the CTT, CGG, GTG, CGT, and GTT haplotypes. The results of the association analysis revealed that the CTT, CGG, GTG, CGT, and GTT haplotypes increased the risk of developing the metabolic syndrome in the research participants by 1.788 (*P* 0.001), 1.622 (*P* 0.033), 1.641 (*P* 0.027), 5.863 (*P* 0.001), and 5.621 (*P* 0.011) times, respectively.

The current case-control study has both strengths and limitations. To the best of our knowledge, this is the first study in Sudan to provide information on the genetic relationship between ADIPOQ variants and susceptibility to the metabolic syndrome risk. The study gathered enough information about lifestyle choices that might be confounding factors in the onset of disease. These variables may also play a role in figuring out how genes and the environment interact.

One limitation of the current study was that no SNPs with proven relationships published in the literature were genotyped due to cost-effectiveness concerns. Based on information from the literature search and their higher allele frequency in the SNP database, the SNPs for the current study were selected.

In conclusion, the results of this study give strong support for the hypothesis that the metabolic syndrome risk in the Sudanese population is strongly influenced by the ADIPOQ-11377C > G (rs266729), +45T > G (rs2241766), and +276G > T (rs1501299) polymorphisms and their haplotype combinations. Given the significance of gene-gene interactions, more studies are required to determine the substantial contributions of ADIPOQ gene variants to the emergence of obesity and other characteristics of metabolic diseases to clarify the pathological processes.

## Figures and Tables

**Table 1:** Details of primers and restriction enzymes used in the study.

Genotypes/SNPs	Primer sequences	Annealing temperature (°C)	Amplicon size (bp)	Restriction enzymes	Digested products (bp)
(rs266729)	FP: 5ʹACTTGCCCTGCCTCTGTCTG-3ʹ	63.0	250	HhaI	137 + 113
11377C > G	RP: 5ʹCCTGGAGAACTGGAAGCTG-3ʹ				

(rs2241766)	FP: 5ʹGTGTGTTGTGGGGTCT3ʹ	56.8	305	SmaI	202 + 103
45T > G	RP: 5ʹTGTGATGAAAGAGGCCAGAA-3ʹ				

(rs1501299)	FP:5ʹCTACACTGATATAAACTATATGGAG3ʹ	59.4	413	BsmI	270 + 143
276G > T	RP: 5ʹCCCCAAATCACTTCAGGTTG-3ʹ				

FP: forward primer; RP: reverse primer; bp: base pair.

**Table 2:** Comparison of anthropometric measurements, blood pressure, HbA1c, and the lipid profile between MetS patients and non-MetS controls.

Variables	Men (*n* = 154)			Women (*n* = 266)		
	MetS (*n* = 77)	Non-MetS (*n* = 77)	*P* value	MetS (*n* = 133)	Non-MetS (*n* = 133)	*P* value
	Mean (SD)^#^			Mean (SD)^#^		
Age (year)	55.1 ± 7.6	54.3 ± 7.2	0.526	53.4 ± 8.4	53.6 ± 6.9	0.813
BMI (Kg/m^2^)	30.77 ± 5.3	23.06 ± 3.5	0.000^*∗*^	31.33 ± 5.0	23.91 ± 4.2	0.000^*∗*^
WC (cm)	111.40 ± 9.1	87.84 ± 12.2	0.000^*∗*^	108.50 ± 11.5	84.00 ± 12.1	0.000^*∗*^
SBP (mmHg)	133.58 ± 13.1	118.70 ± 4.3	0.000^*∗*^	135.75 ± 16.1	118.79 ± 3.8	0.000^*∗*^
DBP (mmHg)	85.84 ± 6.9	79.74 ± 3.1	0.000^*∗*^	85.00 ± 7.3	79.73 ± 2.7	0.000^*∗*^
HbA1c (%)	8.74 ± 2.0	5.64 ± 0.5	0.000^*∗*^	8.84 ± 2.3	5.80 ± 0.4	0.000^*∗*^
TC (mg/dL)	174.18 ± 31.5	171.03 ± 26.0	0.499	189.13 ± 32.2	177.93 ± 28.0	0.003^*∗*^
TG (mg/dL)	154.31 ± 46.4	109.27 ± 32.6	0.000^*∗*^	167.50 ± 56.4	110.05 ± 26.5	0.000^*∗*^
LDL-C (mg/dL)	102.86 ± 31.3	94.27 ± 24.8	0.061	116.07 ± 32.3	92.88 ± 29.0	0.000^*∗*^
HDL-C (mg/dL)	41.03 ± 14.8	52.01 ± 10.5	0.000^*∗*^	47.23 ± 12.9	56.06 ± 8.1	0.000^*∗*^

Data are presented as mean values with SD. MetS: metabolic syndrome, BMI: body mass index, WC: waist circumference, SBP: systolic blood pressure, DBP: diastolic blood pressure, TG: triglyceride, TC: total cholesterol, LDL-C: low-density lipoprotein-cholesterol, and HDL-C: high-density lipoprotein-cholesterol. An independent *t* test was applied. Statistical significance is defined as a two-tailed *P* value ≤0.05.

**Table 3:** Comparison of the genotypes and allele frequencies of the ADIPOQ gene polymorphisms (rs266729, rs2241766, and rs1501299) in MetS patients and non-MetS control.

Gene polymorphisms	Group		*P* value	OR	CI lower-upper
	MetS	Non-MetS			
ADIPOQ rs266729					
Genotypes					
CC	149 (71.0%)	175 (83.3%)	Reference		
CG	48 (22.8%)	32 (15.2%)	0.026^*∗*^	1.762	(1.070–2.899)
GG	13 (6.2%)	3 (1.5%)	0.012^*∗*^	5.089	(1.423–18.20)
Alleles					
C	346 (82.4%)	382 (91.0%)	Reference		
G	74 (17.6%)	38 (9.0%)	0.000^*∗*^	2.150	(1.416–3.264)
ADIPOQ rs2241766					
Genotypes					
TT	142 (67.6%)	174 (82.8%)	Reference		
TG	58 (27.6%)	34 (16.2%)	0.002^*∗*^	2.090	(1.296–3.371)
GG	10 (4.8%)	2 (1.0%)	0.021^*∗*^	6.127	(1.321-28.42)
Alleles					
T	342 (81.4%)	382 (91.0%)	Reference		
G	78 (18.6%)	38 (9.0%)	0.000^*∗*^	2.293	(1.515–3.470)
ADIPOQ rs1501299					
Genotypes					
GG	76 (36.2%)	121 (57.6%)	Reference		
GT	101 (48.1%)	82 (39.0%)	0.001^*∗*^	1.961	(1.303–2.952)
TT	33 (15.7%)	7 (3.4%)	0.000^*∗*^	7.506	(3.161–17.82)
Alleles					
G	253 (60.2%)	324 (77.1%)	Reference		
T	167 (39.8%)	96 (22.9%)	0.000^*∗*^	2.228	(1.650–3.008)

Data are reported as numbers and percentages (%) for genotype counting across all participants (the metabolic syndrome group and control group). Analyses were conducted using the equation of allelic frequencies. A chi-squared test was performed. A two-tailed *P* value ≤0.05 is considered significant. OR: odd ratio, CI: confidence interval.

**Table 4:** Comparison between ADIPOQ (rs266729, rs2241766, and rs1501299) gene polymorphisms and metabolic syndrome components in MetS patients.

Genotypes	BMI (kg/m^2^)	WC (cm)	SBP (mmHg)	DBP (mmHg)	HbA1c (%)	TG (mg/dL)	TC (mg/dL)	LDL-C (mg/dL)	HDL-C (mg/dL)
ADIPOQ rs266729									
CC	30.79 ± 4.7	109.3 ± 11.1	134.0 ± 15.4	85.0 ± 7.6	8.66 ± 2.0	161.1 ± 53.6	182.6 ± 34.4	109.1 ± 34.6	45.1 ± 14.1
CG	32.03 ± 6.1	109.2 ± 10.4	137.7 ± 14.7	85.5 ± 6.4	9.16 ± 2.4	161.3 ± 46.5	186.6 ± 29.6	117.7 ± 26.7	45.4 ± 13.0
GG	31.60 ± 5.4	113.5 ± 7.8	135.8 ± 11.1	86.1 ± 5.0	9.22 ± 2.5	185.0 ± 69.8	184.1 ± 22.6	112.7 ± 25.2	40.4 ± 15.4
*P* value	0.335	0.393	0.329	0.833	0.303	0.297	0.758	0.278	0.492
ADIPOQ rs2241766									
TT	30.99 ± 5.3	109.6 ± 10.8	134.2 ± 14.7	85.0 ± 6.2	8.70 ± 2.2	156.2 ± 51.1^a^	182.9 ± 33.2^a^	109.7 ± 31.2^a^	46.0 ± 14.5
TG	31.22 ± 4.7	109.1 ± 10.8	136.9 ± 16.3	85.8 ± 9.4	9.07 ± 1.9	170.1 ± 54.6^b^	181.5 ± 32.5^b^	109.9 ± 33.4^b^	42.8 ± 12.8
GG	32.31 ± 5.9	110.2 ± 11.5	133.5 ± 11.5	84.5 ± 4.9	8.80 ± 2.2	210.8 ± 50.4	206.1 ± 17.9	141.4 ± 34.0	40.6 ± 9.3
*P* value	0.729	0.936	0.512	0.778	0.558	0.003^*∗*^	0.081	0.011^*∗*^	0.210
ADIPOQ rs1501299									
GG	30.78 ± 5.5	110.1 ± 10.3	134.4 ± 14.6	85.7 ± 7.1	8.54 ± 1.9	158.1 ± 45.6^d^	178.6 ± 29.5^d^	109.5 ± 32.0^d^	43.6 ± 13.4
GT	31.34 ± 4.6	109.4 ± 10.7	133.5 ± 13.4^c^	84.8 ± 7.2	8.94 ± 2.3	159.6 ± 50.2^c^	183.2 ± 31.9^c^	108.6 ± 32.1^c^	45.8 ± 13.8
TT	31.21 ± 5.8	108.7 ± 12.4	140.4 ± 19.5	85.4 ± 7.4	9.00 ± 2.2	182.5 ± 72.6	196.2 ± 39.4	123.4 ± 33.1	44.8 ± 15.6
*P* value	0.771	0.789	0.069	0.691	0.422	0.064	0.035^*∗*^	0.064	0.569

Data are presented as mean value ± SD. MetS: metabolic syndrome, BMI: body mass index, WC: waist circumference, SBP: systolic blood pressure, DBP: diastolic blood pressure, TG: triglyceride, TC: total cholesterol, LDL-C: low-density lipoprotein-cholesterol, HDL-C: high-density lipoprotein-cholesterol. The one-way ANOVA test was used. Two-tailed *P* value ≤0.05 was considered statistically significant. ^a^TT vs. GG *P* value <0.05. ^b^TG vs. GG *P* value <0.05. ^c^GT vs. TT *P* value <0.05. ^d^GG vs TT *P* value <0.05.

**Table 5:** Univariate and multivariate logistic regression analysis of risk factors affecting MetS patients.

Variables	Univariate		^#^Multivariate	
	*P* value	OR (95% CI)	*P* value	OR (95% CI)
Age (year)	0.856	1.002 (0.977–1.028)	—	—
Gender (M/F)	1.000	1.000 (0.672–1.487)	—	—
Hypertension	0.000^*∗*^	11.81 (6.331–22.05)	0.006^*∗*^	8.50 (1.870–38.71)
Hyperglycemia	0.000^*∗*^	17.62 (10.33–30.05)	0.000^*∗*^	24.43 (7.213–82.78)
BMI (kg/m^2^)	0.000^*∗*^	1.469 (1.367–1.579)	0.003^*∗*^	1.254 (1.078–1.458)
WC (cm)	0.000^*∗*^	1.191 (1.154–1.230)	0.000^*∗*^	1.210 (1.134–1.292)
TG (mg/dL)	0.000^*∗*^	1.051 (1.040–1.063)	0.000^*∗*^	1.055 (1.032–1.079)
TC (mg/dL)	0.006^*∗*^	1.009 (1.003–1.016)	0.838	0.997 (0.969–1.026)
LDL-C (mg/dL)	0.000^*∗*^	1.020 (1.013–1.027)	0.643	0.994 (0.967–1.021)
HDL-C (mg/dL)	0.000^*∗*^	0.930 (0.911–0.949)	0.266	0.971 (0.923–1.022)
ADIPOQrs266729				
CG	0.026^*∗*^	1.762 (1.071–2.898)	0.747	1.222 (0.361–4.133)
GG	0.012^*∗*^	5.089 (1.423–18.20)	0.451	4.920 (0.078–310.7)
ADIPOQrs2241766				
TG	0.002^*∗*^	2.090 (1.296–3.371)	0.027^*∗*^	4.454 (1.184–16.78)
GG	0.021^*∗*^	6.127 (1.321–28.42)	0.647	0.467 (0.018–12.11)
ADIPOQrs1501299				
GT	0.001^*∗*^	1.961 (1.303–2.952)	0.039^*∗*^	3.021 (1.056–8.645)
TT	0.000^*∗*^	7.506 (3.162–17.82)	0.383	2.136 (0.388–11.76)

OR: odd ratio, CI: confidence interval. Logistic regression was used for analysis. *P* value ≤0.05 was considered statistically significant. ^#^All variables with *P* value <0.05 were included in the multivariate.

**Table 6:** Haplotype frequencies in the MetS patients and the non-MetS group.

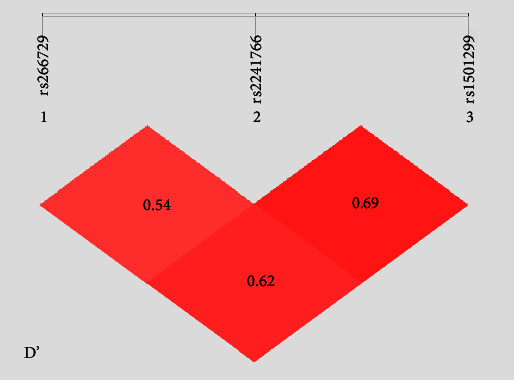				

Linkage disequilibrium analysis of ADIPOQ SNPs				
Haplotypes rs266729, rs2241766, and rs1501299	MetS patients frequency (%)	Non-MetS group frequency (%)	*P* value	Odd ratio (95% CI: lower–upper)

CTG	136 (32.3%)	253 (60.2%)	<0.0001	0.316 (0.238–0.419)
CTT	139 (33.0%)	91 (21.6%)	<0.0001	1.788 (1.313–2.434)
CGG	54 (12.8%)	35 (8.3%)	0.033	1.622 (1.036–2.542)
GTG	56 (13.3%)	36 (8.5%)	0.027	1.641 (1.054–2.554)
CGT	17 (4.0%)	3 (0.7%)	0.001	5.863 (1.705–20.161)
GTT	11 (2.6%)	2 (0.4%)	0.011	5.621 (1.238–25.516)

OR: odd ratio, CI: confidence interval. The genetic analysis identifies a haplotype at the adiponectin locus. *P* value ≤0.05 was considered statistically significant.

## Data Availability

The datasets used and/or analyzed during the current study can be found at the following link: https://figshare.com/articles/dataset/ADIPOQ_gene_data/21687407.
